# Neurological sequelae after childhood bacterial meningitis

**DOI:** 10.1007/s00431-024-05788-w

**Published:** 2024-09-30

**Authors:** Laura Lempinen, Riste Saat, Sakke Niemelä, Anu Laulajainen-Hongisto, Antti A. Aarnisalo, Tea Nieminen, Jussi Jero

**Affiliations:** 1 HUS Medical Imaging Center, Radiology, University of Helsinki and Helsinki University Hospital, PB 340, 00029 HUS Helsinki, Finland; 2 Head and Neck Surgery, Otorhinolaryngology, University of Helsinki and Helsinki University Hospital, Helsinki, Finland; 3 Radiology, East Tallinn Central Hospital, Tallinn, Estonia; 4 Otorhinolaryngology, University of Turku, Turku, Finland; 5 Children’s Hospital, Paediatrics, University of Helsinki and Helsinki University Hospital, Helsinki, Finland; 6 University of Helsinki and Helsinki University Hospital, Helsinki, Finland

**Keywords:** Bacterial meningitis, Child, Neurological sequelae, Death, Hearing loss, Deafness

## Abstract

The purpose of this study is to evaluate childhood bacterial meningitis (BM): incidence, clinical presentation, causative pathogens, diagnostics, and outcome (neurological sequelae, hearing loss, and death). A retrospective review of all children aged ≤ 16 years and 1 month diagnosed with BM at a tertiary children’s centre in the period 2010–2020. The Glasgow Outcome Scale (GOS) was used to assess outcome, with a GOS score of 1–4 considered to be an unfavourable outcome. Logistic regression univariate analysis was used to determine predefined risk factors for death, unfavourable outcome, and long-term neurological sequelae. Seventy-four patients (44 males) with a median age of 8.0 months (range 1 day to 16 years and 1 month) and 77 BM episodes were included in the study. The average incidence rate of BM was 2.2/100,000/year, the majority (91%) being community-acquired BM. *Streptococcus pneumonia* and *Neisseria meningitidis* were the most common pathogens 12/77 (16%) each. Neurological sequelae at discharge were present in 24 (34%) patients, unfavourable outcome in 19 (25%), and hearing loss (deafness) in two (3%) survivors of BM. Seven (9%) patients died. Long-term neurological sequelae were observed in 19/60 (32%), aphasia/dysphasia being the most common in 10 (17%) BM children. No independent risk factors were identified for long-term neurological sequelae in univariate analysis.

*Conclusion:* The risk for a fatal course of BM is still remarkable. Neurological sequelae persisted in a substantial proportion of BM survivors in long-term follow-up, aphasia/dysphasia being the most common. Hearing loss (deafness) occurred in 3%. However, no specific risk factors predicting the long-term sequelae were found.

**Table:** 

**What is Known:** *• Streptococcus pneumonia and Neisseria meningitidis were the most common pathogens causing bacterial meningitis.* *• Risk for fatal course of bacterial meningitis (BM) remains remarkable despite advances in modern medicine.*
**What is New:** *• In long-term follow-up, 1/3 of BM children suffered from neurological sequelae in the 2010s, aphasia and dysphasia being the most common sequelae.* *• Hearing loss was diagnosed in only two (3%) children, whom of both were deaf.*

**What is Known:**

*• Streptococcus pneumonia and Neisseria meningitidis were the most common pathogens causing bacterial meningitis.*

*• Risk for fatal course of bacterial meningitis (BM) remains remarkable despite advances in modern medicine.*

**What is New:**

*• In long-term follow-up, 1/3 of BM children suffered from neurological sequelae in the 2010s, aphasia and dysphasia being the most common sequelae.*

*• Hearing loss was diagnosed in only two (3%) children, whom of both were deaf.*

## Introduction

Bacterial meningitis (BM) remains life-threatening and causes significant morbidity despite improvements in modern medicine [1]. In high-income countries, the fatality rate is approximately 3–10% [2–5] and survivors of BM have a high risk for long-term disabling neurological sequelae, with estimates varying from 20 to 50% [5–7]. The most frequent neurological sequelae after BM are hearing loss, motor deficit, seizures, and cognitive deficits/mental retardation [5–8]. In several studies, the long-term disabling sequelae and hearing loss are associated with pneumococcal meningitis [5, 6, 9].

To prevent BM, many industrialized countries have introduced vaccines against the main pathogens of BM (*Haemophilus influenzae* type b*, Streptococcus pneumoniae*, and *Neisseria meningitidis*) into their vaccination programmes. These conjugate vaccines have had an impact on the bacterial aetiology of BM [10].

Our aim was to evaluate childhood bacterial meningitis (BM): its incidence, clinical presentation, causative pathogens, diagnostics, and outcome (neurological sequelae, hearing loss, and death).

## Materials and methods

### Study design

A retrospective observational cohort study was conducted at the Department of Children and Adolescents at Helsinki University Hospital, which provides healthcare services to a population of approximately 1.7 million people. The study population included all children confirmed with BM from 1 January 2010 to 31 December 2020. Helsinki University Hospital also serves other areas in Finland as a tertiary referral centre, and seven patients came from outside the Helsinki University Hospital district. At the time of the study, the catchment population < 16 years of age in Helsinki and Uusimaa Hospital District (HUS) increased from 278,320 in 2010 to 295,525 in 2020. Over an 11-year period, the mean catchment population was 288,637, which was used to calculate the BM incidence (BM cases/100,000/year). Seven patients from outside our hospital district were excluded from the BM incidence calculation.

Patient data were obtained from an electronic database. The International Classification of Diseases (ICD) version 10 was used for data search, using the following codes: G00.0, G00.1, G00.2, G00.3, G00.8, G00.9, G01*, G01*A01.0, G01*A02.2, G01*A17.0, G01*A32.1, G01*A39.0, G03, G03.0, G03.1, G03.2, G03.8, G03.9, A39.0, G01*A22.8, G05.0*A17.8, G05.0*A32.1, G05.0*A39.8. A total of 178 patients’ electronic medical records were assessed to obtain demographic information, clinical presentation, laboratory data, imaging, microbiological data, treatment (medical and surgical), hospital course, and outcomes.

Patients were included in the study if they had signs and symptoms suggestive of BM and met at least one of the following criteria: (1) positive cerebrospinal fluid (CSF) culture, (2) detection of bacterial DNA or antigens in CSF, (3) detection of bacteria in CSF Gram stain. (4) CSF leukocytosis > 53 × 10^6^/L and CSF protein > 473 mg/L were together cut point values, if CSF culture, DNA/antigen, or CSF gram stain was negative in patients having signs and symptoms suggestive of BM.

CSF sampling failed with one newborn (age < 72 h) diagnosed with clinical meningitis: the blood culture was positive for group B streptococcus (GBS), CRP was 50 mg/L, and plasma leucocyte count was 7.7 E9/l. In addition, two cases of tuberculous meningitis with a positive B-LyTbIFN/Tb-Spot test were included in the study.

Patients with meningitis caused by borrelia or non-bacterial pathogens (virus, fungi, or parasite) were excluded.

Community-acquired BM (CABM) was defined as ≥ 3 days of age, BM acquired out of hospital, and no neurosurgery within the previous 30 days. Nosocomial BM was defined as symptoms occurring within 48 h after admission to the hospital, 3 days after discharge, or 30 days after neurosurgical operation [11]. BM in a newborn baby with perinatal infection from birth was also considered nosocomial.

*Haemophilus influenzae* type b vaccines have been given in the Finnish national vaccination programme at the ages of 3, 5, and 12 months since 1993. The 10-valent pneumococcal conjugate vaccine (Synflorix^R^, covering *S. pneumoniae* serotypes 1, 4, 5, 6B, 7F, 9V, 14, 18C, 19F, and 23F) has been given with the same schedule since the autumn of 2010. Meningococcal vaccines are not included in the Finnish national vaccination programme for children.

## Patient data

All magnetic resonance imaging (MRI) and computed tomography (CT) studies of the head and neck region were re-assessed by two radiologists (RS and LL) for neuroradiological findings and complications. The radiologists’ reports of brain ultrasound studies were also inspected for intracranial (IC) complications. The following conditions were defined as neuroradiological complications: haemorrhage; infarction; presence of newly acquired or acerbation of hydrocephalus; venous infarction; pus in subdural, subarachnoidal space, or lateral ventricles; unknown subdural effusion/hygroma; brain herniation; brain oedema; luminal narrowing of the internal carotid artery; encephalitis; spinal epidural abscess; and labyrinthitis.

Hearing tests were done on 47/74 BM children (64%). Hearing was tested by otoacoustic emissions in 38 patients, by audiogram in seven, by behavioral observation audiometry in three, and by brainstem-evoked response audiometry in one patient. Hearing loss was defined as > 25 decibels Hearing Level (dB HL) and further subdivided into moderate (41–60 dB HL), severe (61–79 dB HL), and profound (deaf) (≥ 80 dB HL). We present the hearing thresholds according to the better-ear hearing level and by each ear separately (ear-specific).

Patients were categorized according to the Glasgow Coma Scale (GCS). If GCS data collected from patient records was incomplete, the level of consciousness was estimated based on the available information. Coma was defined as need for intubation or moderately to severely altered mental status (3–8 points), altered level of consciousness as mildly altered mental status (9–12 points), and normal level of consciousness (13–15 points). The Glasgow Outcome Scale (GOS) was used to assess outcome at discharge: death (1 point), vegetative state (2 points), severe disability (3 points), moderate disability (4 points), and mild or no disability (5 points) [12]. An unfavourable outcome was defined as a GOS score of 1–4.

Long-term neurological sequelae were defined as persistent sequelae after discharge, diagnosed during hospital stay or follow-up, including hearing loss/deafness with cochlea implant, seizure disorder, dizziness, dysphasia/aphasia, isolated hypotonia/motor delay, and learning difficulties. Seizure disorder was defined as children having seizures and/or receiving prophylactic anti‐epileptic drugs during follow‐up, excluding patients who stopped such treatment at their first routine outpatient appointment after BM. Follow‐up time ranged from 2 months to 9 years and 3 months. Twenty-seven patients had less than 12 months of follow-up in specialized healthcare. After specialized healthcare follow-up, monitoring continued at child welfare clinics for preschoolers and school health services for school-aged children.

## Statistical analyses

Statistical analysis of the data was conducted in IBM SPSS v29. Fisher’s or *χ*2 exact test when appropriate was used to determine significant differences between categorical variables. The Mann–Whitney *U* test was used to analyze the correspondence of medians with the interquartile ranges (IQR) or range of continuous variables. Univariate logistic regression analysis was used to predict death, an unfavourable outcome, or long-term neurological sequelae. The results are expressed as odds ratios (OR) with a 95% confidence interval (CI_95%_). A two-tailed *P*-value of < 0.05 was considered statistically significant.

## Results

**Fig. 1:**
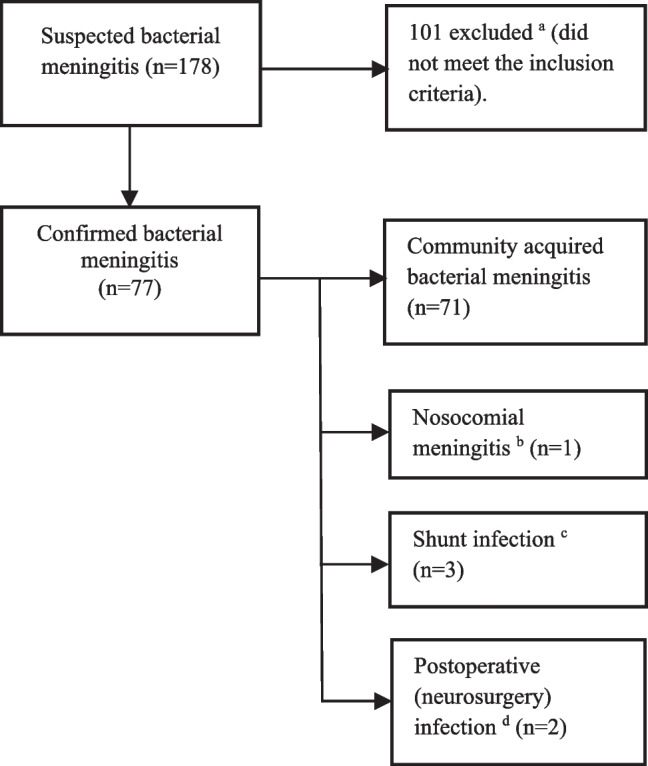
Study flow chart. ^a^Meningitis caused by borrelia, or non-bacterial pathogens (virus, fungi, or parasite). ^b^BM in a newborn baby with perinatal infection from the birth was considered as nosocomial. ^c^Intracranial malignant and benign tumour in one each. ^d^Bacterial meningitis diagnosed < 30 days after neurosurgery

**Fig. 2:**
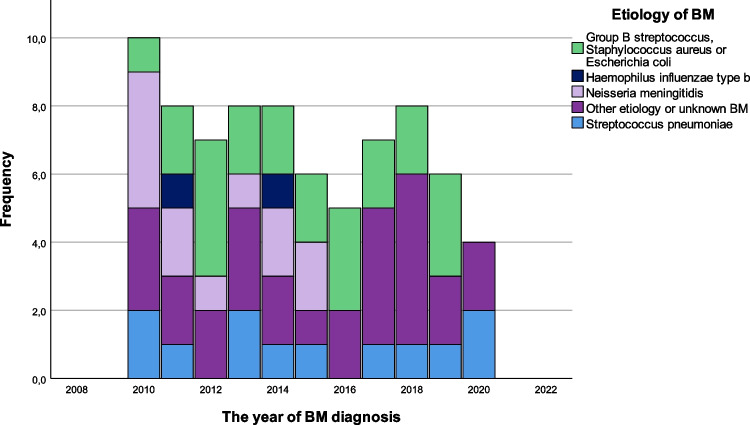
Incidence rates and aetiology of childhood bacterial meningitis in Southern Finland, 2010–2020. Other aetiology included *Mycobacterium tuberculosis* in 2, and gram-positive cocci, *Pseudomonas aeruginosa*, *Staphylococcus epidermis*, and *Staphylococcus hominis* in one each. Unknown aetiology in 22

Seventy-seven BM episodes from 74 patients were included in this study (Fig. 1). The median age at diagnosis was 8.0 months (range from 1 day to 16 years and 1 month), and 44 (57%) were males. The incidence of BM in our hospital district was approximately 2.2/100,000/year. The majority (71 (92%)) were community-acquired BM (CABM). The incidence rates and aetiology of childhood bacterial meningitis per year are shown in Fig. 2. Table 1 summarizes the baseline characteristics of BM children.

**Table 1:** The baseline characteristics of children diagnosed with bacterial meningitis, 2010–2020

	All *N* = 77 *n*/*N* (%) or median (IQR)	Children aged < 2 months old *N* = 24 *n*/*N* (%) or median (IQR)	Children aged ≥ 2 months old *N* = 53 *n*/*N* (%) or median (IQR)
Demographics			
Age, months	8,0 (1.3–55)	0.8 (0.3–1.3)	19.0 (6.0–119.0) ^<*0.0001*^
Sex (males)	44 (57)	13 (54)	31 (58)
Predisposing infection^a^	34 (44)	10 (42)	24 (45)
Prior hospital admission			
Upper respiratory infection symptoms	22 (29)	-	22 (42) ^<*0.0001*^
Duration of symptoms, days	1 (1–3)	1 (1–1)	2 (1–5) ^<*0.0001*^
Previous antibiotic	19 (25)	3 (13)	16 (30)
Nausea/vomiting	32/75 (43)	3/23 (13)	29/52 (56) ^*0.001*^
Petechia	4/74 (5)	-	4/51 (8)
Seizures	4/75 (5)	-	4/51 (8)
Altered level of consciousness	11/75 (15)	1/23 (4)	10/52 (19)
Coma	2/75 (3)	-	2/52 (4)
Clinical and laboratory findings on admission			
Headache (age ≥ 16 months)	11/28 (39)	-	11/28 (39)
Nausea/vomiting	13/72 (18)	2/22 (9)	11/49 (22)
Meningeal irritation	16/73 (22)	-	16/49 (33) ^*0.001*^
Petechiae	13/71 (18)	2/23 (9)	11/48 (23)
Fever > 37.9 °C	36/50 (72)	12/14 (86)	24/36 (67)
Irritable (age ≤ 9 months	22/37 (59)	12/21 (57)	10/16 (63)
Dyspnoea (*n* = 68)	10/68 (15)	4/21 (19)	6/47 (13)
Seizures	5/74 (7)	1 (4)	4/50 (8)
Triad (fever, altered level of consciousness, and meningeal irritation) (*n* = 66)	9/66 (14)	-	9/47 (19) ^*0.035*^
Altered level of consciousness	21/74 (28)	6 (25)	15/50 (30)
Coma	7/74 (9)	3 (13)	4/50 (8)
Any neurological sequelae^b^	21/74 (28)	3 (13)	18/50 (36) ^*0.036*^
Cranial nerve palsy^c^	3/70 (4)	0/22 (0)	3/48 (6)
B-Leukocytes (× 10^9^), day1 (*n* = 75)	12.0 (5.9–19.6)	6.7 (3.5–13.3)	15.1 (8.1–22.3)^*0.003*^
C-reactive protein (mg/L) day1 (*n* = 75)	90 (45–189)	82.5 (33.5–99.25)	97 (53–199)
Blood culture positive (*n* = 69)	47 (61)	18 (75)	29/45 (64)
CSF leucocyte count (× 10^6^/L) day1 (*n* = 70)	1359 (213–2923)	1920 (592–3028)	1130 (125–3200)
CSF protein (mg/L) (*n* = 66)	1706 (985–2753)	1846.5 (1226–2435)	1291(702–3140)
CSF erythrocytes (× 10^6^/L) (*n* = 68)	161 (30–697)	418 (54–3310)	180 (17–660) ^*0.033*^
CSF glucose (mmol/L) (*n* = 63)	2.3 (0.9–3.6)	1.85 (1.2–2.5)	2.9 (1.0–4.1)
CSF culture detection of pathogen^d^	55/76 (72)	15 (63)	40 (75)
Blood culture positive	48/70 (69)	18/23 (78)	30/47 (64)
During hospital stay			
Coma	13 (17)	3 (13)	10 (19)
Seizures	18 (23)	10 (42)	8 (15) ^*0.025*^
Any neurological sequelae^b^	46 (60)	14 (58)	32 (60)
Intensive care unit admission	26 (34)	6 (25)	20 (38)
Adjunctive anticonvulsant treatment	14 (18)	7 (29)	7 (13)
Operative treatment	12 (16)	-	12 (23) ^*0.014*^
Neuroradiological complication	37/66 (56)	13/23 (57)	24/44 (55)

^a^Urinary tract infection in 17, otitis media/acute mastoiditis in 8, sinusitis in 6, pneumonia and arthritis in 2 each, and skin infection, and pleural empyema in one each

^b^Defined as seizures, dizziness, cranial nerve palsy, hemiparesis, aphasia/dysphasia, isolated hypotonia/motor delay, ataxia, any psychomotor retardation, hearing impairment, papillary stasis, hydrocephalus, or memory difficulties

^c^Ptosis, abducens paresis, and strabismus in one

^d^Includes patients diagnosed by CSF with positive CSF culture (*n* = 44), non-culture detection of bacterial DNA or antigen in CSF (*n* = 8), and detection of bacteria on CSF Gram stain (*n* = 1). Two mycobacterium tuberculosis meningitis with positive B-LyTbIFN/Tb-Spot test. One lumbar puncture failed

Statistical significance when *P* < 0.05.

## Medical history, symptoms, clinical findings, microbiology, and laboratory tests

Twenty out of 77 (26%) children had underlying chronic diseases: hydrocephalus (*n* = 3); intracranial tumour (malignant and benign); asthma; newborn asphyxia; human immunodeficiency virus positivity; neonatal incontinentia pigmentia gravis; megalencephaly capillary malformation syndrome; sensorineural and conductive (combined) hearing impairment; Down syndrome with duodenal stenosis; lumbalic hemivertebra; neurofibromatosis type I and middle cerebral artery infarction; cheilopalatoschisis and anetoderma of Jadassohn-Pelliazari.

Nine infants aged < 2 months were born preterm before 37 + 0 weeks of gestation: two before 27 + 0 weeks and the rest between 29 + 2 weeks and 35 + 5 weeks. Eight mothers tested positive for GBS. Five children had a neurosurgical operation before BM diagnosis. Four children had had BM before.

At admission, fever (> 38 °C) was present in 36/50 (72%), headache in 11/28 (39%), altered level of consciousness in 21/74 (28%), any psychomotor retardation in 14/71 (20%), petechia in 13/71 (18%), nausea/vomiting in 13/72 (18%), and seizures in 5/74 (7%). The median C-reactive protein (CRP) level at admission was 90 mg/L (IQR 45–189). During hospital treatment, the maximum median CRP in children ≥ 2 months old (245, IQR 153–305) was higher compared with infants < 2 months old (130, IQR 72–204; *P* = 0.004). Disseminated intravascular coagulation was diagnosed in six children ≥ 2 months old. The most common pathogen causing BM in children ≥ 2 months old was *N. meningitidis* (*n* = 12, 23%; *P* = 0.20), followed by *S. pneumoniae* (n = 11, 21%), and in children < 2 months old it was GBS (*n* = 11, 46%; *P* < 0.001) (Table 2). Of 21 patients with negative CSF culture, 14 (67%) had pre-diagnostic antibiotic therapy.

**Table 2:** Aetiology of bacterial meningitis in children in Southern Finland, 2010–2021

Pathogen	All *N* = 77 (%)	Children aged < 2 months old *N* = 24 (%)	Children aged ≥ 2 months old *N* = 53 (%)
Unknown causative agent	22 (29)	9 (39)	13 (14)
*Neisseria meningitidis*	12 (16)	-	12 (23) ^*0.020*^
*Streptococcus pneumonia*	12 (16)	1 (4)	11 (21)
*Haemophilus influenzae type* b	2 (3)	-	2 (4)
Group B *streptococcus* (GBS)	13 (17)	11 (46)	2 (4) ^<*.0001*^
*Staphylococcus aureus*	6 (8)	1 (4)	5 (9)
*Escherichia coli*	4 (5)	2 (8)	2 (4)
*Pseudomonas aeruginosa*	1 (1)	-	1 (2)
*Staphylococcus epidermis*	1 (1)	-	1 (2)
*Staphylococcus hominis*	1 (1))	-	1 (2)
Gram positive cocci	1 (1)	-	1 (2)
*Mycobacterium tuberculosis*	2 (3)	-	2 (4)

Statistical significance when *P* < 0.05.

### Operative treatment, neuroradiological complications, and hearing results

A total of 16 operations were performed on 12 children within a median of 6.5 days (IQR 1.5–64) after diagnosis of BM. In four children, the operation was performed after discharge. The most common operation was an IC shunt operation/trepanation in seven, followed by tympanostomy in three, cochlear implantation and paranasal sinus surgery in two each, and application of ICP-monitoring (Codman) and revision of distal necrosis of fingers and toes in one each. The cochlear implantation was performed on average 2.5 months after the diagnosis. Twenty-six (34%) children were admitted to an intensive care unit. Adjunctive anticonvulsant treatment was administered to 14 (18%) patients and empiric adjunctive corticosteroid treatment to 26 (34%) patients.

Neuroradiological complication occurred in 37 out of 66 (56%) scanned patients. The most common findings were pus in subdural, subarachnoidal space or in lateral ventricles (*n* = 15), the presence of newly acquired or acerbation of hydrocephalus (*n* = 10), haemorrhage (*n* = 9), unknown subdural effusion/hygroma (*n* = 7), infarction (*n* = 6), labyrinthitis (*n* = 4), and venous infarction and brain herniation in three each. Brain oedema, encephalitis, narrowing of ICA lumen, and spinal epidural abscess were seen in one each. Haemorrhage was diagnosed (30% (7/23)) more often in infants < 2 months old compared with (5% (2/44), *P* = 0.006) children ≥ 2 months old.

Table 3 shows the hearing results. Hearing was tested mainly after hospitalization in 49 out of 77 patients (62%); only one child was tested during hospital stay (3 days after arrival). The initial hearing test was done at a median of 1.8 months after BM diagnosis (IQR 3 weeks to 2 months). Forty-seven out of 49 (96%) children were diagnosed as having normal hearing, and two children were diagnosed with deafness. Possible fluctuation in hearing levels was seen in 5/49 (10%) children.

**Table 3:** The hearing results in children with BM

	All children (%)
The number of children tested for hearing	49/77 (64)
Possible fluctuations in hearing levels after BM	5/49 (10)
The final hearing according to BEHL (*N* = 49)	
Hearing normal	47/49 (96)
Hearing loss (26–79 dB HL)	-
Deaf	2/49 (4)
The final hearing thresholds, ear-specific (*N* = 98)	
Hearing normal	92/98 (94)
Hearing loss (26–79 dB HL)^a^	1/98 (1)
Deaf^b^	5/98 (5)

*BM* bacterial meningitis, *BOA* behavioral observation audiometry

*dB*, decibel, *BEHL*, better ear hearing level

^a^The grade of hearing loss unclear (hearing tested by BOA)

^b^*Streptococcus pneumoniae* in four ears, *Neisseria meningitidis* in one ear

## Outcome

Tables 4 and 5 summarize the clinical outcome. The median duration of hospital stay was 13 days (IQR 8–17 days). At discharge, an unfavourable outcome was observed in 19/70 (27%) BM survivors. Seven (9%) children died within 4 days of admission; 85% (6/7) were boys (age range 1.3 months to 15 years and 11 months at the time of death). Of seven deaths, three were due to meningococcal disease, two *S. pneumoniae*, one *S. aureus*, and one GBS.

**Table 4:** Outcome of children diagnosed with bacterial meningitis in Southern Finland, 2010 2020

	All *N* = 77 (%)	Children < 2 months old *N* = 24 (%)	Children ≥ 2 months old *N* = 53 (%)
Any neurological sequelae at discharge (*n* = 70)	24/70 (34)	7/22 (32)	17/48 (35)
Dizziness	9/70 (13)	-	9/48 (19)
Isolated hypotonia/motor delay	9/70 (13)	4/22 (18)	5/48 (10)
Seizure disorder	5/70 (7)	2/22 (9)	3/48 (6)
Cranial nerve palsy^a^	3/70 (4)	1/22 (5)	2/48 (4)
Hearing loss/deafness^b^	2/70 (3)	-	2/48 (4)
Hemiparesis	2/70 (3)	-	2/48 (4)
Any psychomotor retardation	1/70 (1)	-	1/48 (2)
Ataxia	1/70 (1)	-	1/48 (2)
Aphasia/dysphasia	1/70 (1)	-	1/48 (2)
Glasgow Outcome Score at discharge			
1	7 (9)	2 (8)	5 (9)
2	-	-	-
3	7 (9)	2 (8)	5 (9)
4	5 (6)	1 (4)	4 (8)
5	58 (75)	20 (83)	38 (72)
Long-term sequelae (*n* = 60)	19/60 (32)	8/21 (38)	11/39 (28)
Aphasia/dysphasia	10/60 (17)	5/21 (24)	5/39 (13)
Learning difficulties	5/60 (8)	1/21 (5)	4/39 (10)
Isolated hypotonia/motor delay	3/60 (5)	2/21 (10)	1/39 (3)
Hearing loss (deafness *n* = 2)^b^	2/60 (3)	-	2/39 (5)
Seizure disorder	2/60 (3)	2/21 (10)	-
Dizziness	1/60 (2)	-	

^a^Abducens nerve palsy in two, double vision in one

^b^Includes two patients with cochlea implants, both had pneumococcal aetiology

Statistical significance when *P* < 0.05.

**Table 5:** Univariate analysis—the risk factors for GOS1/death, unfavourable outcome, and long-term neurological sequelae

	GOS1/death (*n* = 7)		Unfavourable outcome (*n* = 19)		Long-term neurological sequelae (*n* = 19)	
	OR (95%, CI)	*P*-value	OR (95%, CI)	*P*-value	OR (95%, CI)	*P*-value
Demographics, prior hospital admission						
Age ≥ 2 months	1.15 (0.20–6.37)	0.88	1.97 (0.58–6.74)	0.28	0.80 (0.25–2.49)	0.70
Male	5.05 (0.58–44.19)	0.14	2.61 (0.83–8.20)	0.10	1.48 (0.49–4.52)	0.49
Otitis media/acute mastoiditis or sinusitis	0.0 (0.00–)	1.0	1.14 (0.32–4.11)	0.84	0.51 (0.12–2.10)	0.35
Petechia prior hospital	13.2 (1.52–114.52)	0.019	3.18 (0.42–24.29)	0.27	2.17 (0.13–36.62)	0.59
Altered level of consciousness prior hospital	11.62 (2.15–62.9)	0.004	1.87 (0.48–7.26)	0.37	0.0 (0.0–)	1.0
During hospital admission						
Dyspnoea at admission	24.43 (2.23–268.06)	0.009	2.85 (0.69–11.85)	0.15	0.0 (0.0–)	1.0
Seizures at admission	8.53 (1.15–63.52)	0.036	15.71 (1.63–151.88)	0.017	1–12 (0.10–13.18)	0.93
Any neurological sequelae at admission	7.97 (1.41–45.10)	0.019	5.11 (1.64–15.98)	0.005	0.47 (0.11–1.92)	0.29
Any neurological sequelae at admission + altered level of consciousness at admission	12.27 (1.39–108.30)	0.024	2.64 (0.89–7.82)	0.08	0.27 (0.07–1.09)	0.07
Coma at admission	11.81 (1.94–71.90)	0.007	1.63 (0.27–9.71)	0.59	0.0 (0.0–)	1.0
Pneumococcal aetiology	1.6 (0.27–9.49)	0.61	1.76 (0.47–6.56)	0.40	0.92 (0.19–4.34)	0.91
CSF leucocyte count < 100 (× 10^6^/L)	13.5 (1.29 –151.28)	0.03	1.18 (0.32–5.32)	0.81	1.89 (0.44–8.09)	0.39
During hospital stay						
Coma during hospital stay	54.00 (5.66–515.64)	< 0.001	3.36 (0.96–11.72)	0.057	3.38 (0.67–16.93)	0.14
Normal level of consciousness during hospital stay	0.49 (0.006–0.44)	0.007	0.44 (0.15–1.30)	0.14	1.06 (0.28–3.98)	0.94
Seizures during hospital stay	5.33 (1.07–26.61)	0.041	4.90 (1.56–15.43)	0.007	1.14 (0.63–7.93)	0.21
Any neurological sequelae during hospital stay	1.77 (0.32–9.75)	0.51	8.50 (1.80–40.17)	0.007	1.67 (0.81–8.77)	0.11
Petechia during hospital stay	6.75 (1.25–36.52)	0.027	0.71 (0.14–3.81)	0.71	1.09 (0.18–6.53)	0.93
Neuroradiological complication	25 × 10^8^ (0.0–)	1.0	9.55 (1.97–46.23)	0.005	1.60 (0.50–5.13)	0.43
Infarction	2.85 (0.66–30.62)	0.39	7.39 (1.22–44.88)	0.030	0.50 (0.05–4.85)	0.55
Newly acquired or acerbation of hydrocephalus	13 × 10^8^ (0.0–)	1.0	4.80 (1.23–18.67)	0.024	1.47 (0.22–9.71)	0.69
SD effusion NAS	7.60 (1.02–56.68)	0.048	2.46 (0.49–12.35)	0.27	0.0 (0.0–)	1.0
Adjunctive anticonvulsant treatment	1.93 (0.34–11.17)	0.46	6.30 (1.82–21.83)	0.004	0.77 (0.18–3.31)	0.73
Operative treatment	0 (0–)	1.0	5.06 (1.44–17.79)	0.012	2.57 (0.64–10.27)	0.18
Intensive care unit admission	15.0 (1.70–132.64)	0.015	2.92 (1.002–8.49	0.050	1.11 (0.34–3.62)	0.86

Statistical significance when *P* < 0.05.

At discharge, neurological sequelae occurred in 24 (34%) of 70 survivors, with dizziness as the most common (9/70; 13%). In the follow-up after hospital discharge, long-term neurological sequelae were diagnosed in 19/60 (32%) patients, aphasia/dysphasia being the most common in 10/60 (17%). Hearing loss/deafness was diagnosed in only two (3%) children. The median follow-up was 1 year (IQR 0.3–3.4 years).

Table 4 summarizes the risk factors for death, an unfavourable outcome at discharge, and long-term neurological sequelae. In univariate analysis, a patient diagnosed with petechiae prior to hospital admission (13.2 (1.52–114.52), *P* = 0.019), altered level of consciousness with neurological sequelae on admission (12.27 (1.39–108.30), *P* = 0.024), seizures on admission (8.53 (1.15–63.52), P = 0.036), or seizures during hospital stay (5.33 (1.07–26.61), *P* = 0.041) showed higher odds for death. Also, SD effusion on imaging (7.60 (1.02–56.68), *P* = 0.48) showed higher odds for a fatal outcome.

## Discussion

Our article is an overview of childhood BM in Southern Finland and its prognostic factors for poor outcomes in 2010–2020. We evaluated the incidence rate, bacteriology, predisposing factors, clinical characteristics, and outcome. The overall incidence rate of BM remained quite stable, at an average of 2.2/100,000/year in infants and children.

Increased use of vaccination against the predominant pathogens has decreased the global incidence of childhood BM in developed countries [10]. The leading pathogen of BM in our paediatric cohort among children ≥ 2 months old was *Neisseria meningitidis*, followed by *Streptococcus pneumoniae*. The 10-valent pneumococcal conjugate vaccine was added to the Finnish national vaccination programme in the autumn of 2010, at the beginning of our period under study. In our study, the major causative organism among infants < 2 months of age remained GBS, as previously published [3, 13]. The fatality rate was 9% in our paediatric cohort. All deaths were related to CABM, and our result is in line with what has previously been reported (3–10%) [2–5]. Despite advances in modern medicine, the overall case fatality rate has not significantly decreased in the twenty-first century in the developed world.

In our data, some children experienced neurological symptoms upon discharge from hospital, and some of them were diagnosed with neurological sequelae during follow-up. Long-term neurological sequelae were common in our series, being present in 32% of BM survivors. The long-term neurological sequelae were slightly more common in infants < 2 months old compared with children ≥ 2 months old, 38% vs. 28% respectively. Infants < 2 months old are the most vulnerable age group, with a higher risk for BM sequelae. These results are consistent with previously reported studies and highlight the persistent challenges regarding childhood BM [5, 13, 14].

In our study, the most common long-term neurological sequelae were aphasia/dysphasia, followed by learning difficulties and isolated hypotonia/motor delay. Svendsen et al. found that abnormalities on cranial imaging, pneumococcal aetiology, and seizures during admission were more often related to the long-term neurological sequelae. However, we found no independent risk factors in logistic regression univariate analysis in relation to long-term neurological sequelae. In our cohort, the neuroradiological complications seem to predict an unfavourable outcome in BM at discharge. However, over the long term, a statistically significant association was no longer observed. Pneumococcal aetiology as an independent factor has also previously been associated with increased risk for long-term neurological sequelae [1, 5, 6, 8]. In our study, however, no statistically significant association was found. This might be partly due to the limited sample size in our data.

The prevalence of post-meningitis hearing loss is known [5, 6, 9, 15]. In our cohort, 64% of children underwent hearing assessments, which is less than ideal. This issue needs to be given more attention in the future. Future guidelines must be revised to ensure that hearing is assessed in every individual diagnosed with BM [16]. The incidence of hearing loss (3%) in our study was lower compared with previous reports, where it has been the most common neurological sequela (14–35%) [4–6]. Both children with hearing loss became deaf; less severe hearing loss was not detected. In earlier studies, bilateral severe to profound hearing loss has been reported in approximately 1–5% of BM survivors, and this is in line with 3% in our study [9, 17]. We observed possible transient fluctuation in hearing levels after BM in 10%. In the prospective BM study by Dodge et al., transient hearing loss was found in 16% of children [18].

It is imperative to monitor hearing during BM because associated bacterial labyrinthitis may lead to partial or total ossification of the labyrinth [19], as was the case in the two patients in our study with profound hearing loss. In this case, insertion of the cochlear implant electrode may become very challenging. If cochlear implantation seems necessary, imaging should be performed. The best results with cochlear implants for a deaf ear are obtained when the procedure is performed without significant delay [20]. Hearing loss is usually associated with *Str. pneumonia* [4, 5, 8, 9], as we also found in both patients with profound hearing loss. Karppinen et al. found that *S. pneumoniae* was the most common cause of impaired hearing at a > 60 dB threshold among children with BM, and it caused deafness more often than Hib and *N. meningitidis* [21].

The treatment protocols of BM, early diagnosis, prompt administration of appropriate antimicrobial agents, and comprehensive supportive care may have together contributed to a more favourable outcome in terms of neurological sequelae due to BM. This finding underscores the importance of not only identifying the causative agents of BM but also implementing effective therapeutic strategies to prevent the immediate and long-term consequences of BM. Quick access to treatment is crucial when treating childhood BM. The availability of intensive care, close monitoring, and efficient management of patients when they are having seizures is very important. This also supports spontaneous recovery tendency in childhood BM.

The main limitation in our study was its retrospective nature, but the treatment protocol for BM is considerably standardized in our tertiary centre. Another limitation is our small sample size due to our country’s small population and the low incidence of BM. In addition, 14% of survivors of BM were lost to follow-up. Despite the imaging and auditory monitoring of patients not being entirely comprehensive, we obtained valuable information regarding the aetiology and long-term sequelae of childhood BM.

One of the best ways to prevent BM is to have effective coverage of conjugate vaccines against the main causes of BM. Infants aged < 2 months are the most vulnerable age group, having the greatest risk for long-term BM sequelae. In future, an effective maternal vaccine for GBS is needed for this vulnerable age group [22, 23].

This study highlights the high fatality risk in childhood BM. Furthermore, long-term neurological sequelae persist among BM survivors, with aphasia/dysphasia being the most common. The incidence of profound hearing loss was 3%. Despite the high morbidity of BM, children tend to have a good recovery.

## Data Availability

Study data can be provided upon reasonable request by the corresponding author.

## Abbreviations

AM: Acute mastoiditis
BM: Bacterial meningitis
CABM: Community-acquired bacterial meningitis
CT: Computed tomography
CSF: Cerebrospinal fluid
dB HL: Decibels Hearing Level
GBS: Group B streptococcus
GCS: Glasgow Coma Scale
GOS: Glasgow Outcome Scale
IC: Intracranial
IQR: Interquartile range
Hib: *Haemophilus influenzae* Type b
LO: Labyrinthitis ossification
OM: Otitis media
PCV: Pneumococcal conjugate vaccine
MRI: Magnetic resonance imaging
